# Distinct clinicopathological features of neuroendocrine liver metastases originating from the pancreas and rectum

**DOI:** 10.1186/s12957-024-03476-5

**Published:** 2024-08-03

**Authors:** Hao Zhang, Takahiro Tsuchikawa, Satoshi Takeuchi, Hang Deng, Kimitaka Tanaka, Aya Matsui, Yoshitsugu Nakanishi, Toshimichi Asano, Takehiro Noji, Toru Nakamura, Shintaro Takeuchi, Masataka Wada, Jian Xu, Yu Zhang, Satoshi Hirano

**Affiliations:** 1 Department of Hepatobiliary Surgery, Sichuan Provincial People’s Hospital, School of Medicine, University of Electronic Science and Technology of China, Chengdu, 610072 China; 2 Department of Gastroenterological Surgery II, Hokkaido University Faculty of Medicine, Sapporo City, 060-8638 Japan; 3 Department of Medical Oncology, Faculty of Medicine, Graduate School of Medicine, Hokkaido University, Sapporo City, 060-8638 Japan; 4 School of Medicine, University of Electronic Science and Technology of China, Chengdu, 610054 China

**Keywords:** Neuroendocrine liver metastasis, Clinicopathological feature, Observation rules, Prognosis

## Abstract

**Introduction:**

Survival comparisons among patients with liver metastases from pancreatic and rectal neuroendocrine tumors (NETs) were limited, and the efficacy of observation rules in patients undergoing hepatectomy for neuroendocrine liver metastases (NELMs) was unknown. This study aims to distinguish these characteristics and clarify the effects of the observation rules on NELMs.

**Methods:**

Clinical data were separately collected from patients with pancreatic and rectal NELMs at medical centers in both Japan and China. The Japanese cohort followed the observation rules for the resection of NELMs. A comparative analysis was conducted on clinical characteristics and prognosis features such as overall survival time (OS) and disease-free survival interval (DFS-I).

**Results:**

Enrollment included 47 and 34 patients from Japan and China, respectively. Of these, 69 and 12 patients had tumors originating from the pancreas and rectum, respectively. The OS time in patients undergoing primary tumor resection was significantly longer; however, the OS time between the patients undergoing and not undergoing radical resection of liver metastasis was the same. In asynchronous NELMs, patients with rectal (R)-NELMs showed a significantly higher proportion of type III NELMs. Additionally, the median DFS-I of asynchronous R-NELMs was longer than the recommended follow-up time, with 71.4% of them classified as G2. In the Japanese cohort, patients who adhered to the observation rules exhibited a longer median DFS after hepatectomy for NELMs compared with their counterparts.

**Conclusion:**

Although curative surgery is crucial for primary lesions, personalized approaches are required to manage NELMs. Extended overall follow-ups and shortened follow-up intervals are recommended for G2 stage rectal NETs. The observation rules for NELMs require further validation with a larger sample size.

## Introduction

Neuroendocrine neoplasms (NENs) are rare diseases originating from a broad range of systemic organs, and their incidence has steadily increased in recent years [1, 2]. Most original NENs are small and insidious, making early diagnosis difficult, especially for nonfunctional NENs. Approximately 20% of patients present with distant metastases at initial diagnosis, and another 38% are metachronous [3]. The most common organ of metastasis is the liver, accounting for approximately 45% of all metastases [4]. Benefiting from improvements in treatments, such as new molecular targeting therapy, peptide receptor radionuclide therapy (PRRT), and transarterial radioembolization, the survival rate for all NENs has significantly improved over time [2]. Although the prognosis of advanced-stage diseases with distant metastasis has also improved, the overall survival (OS) is unsatisfactory [3, 5] and the 5-year OS rate of patients with neuroendocrine liver metastases (NELMs) is only 28.6% [4].

According to the latest World Health Organization (WHO) criteria [6], NENs are staged as well-differentiated neuroendocrine tumors (NETs), grade 1, 2, and 3 NETs (G1, G2, and G3 NETs), and poorly differentiated neuroendocrine carcinomas (NECs). Both NEC and NET patients may be complicated with NELMs throughout the disease course. However, in accordance with the 2023 European Neuroendocrine Tumor Society (ENETS) guidelines, systemic treatment or observation is recommended first for NELMs, and hepatectomy is only considered when systemic treatment is ineffective [7]. However, NELMs significantly weaken the survival benefit of systemic treatment [8–10]. This is a significant challenge for the traditional “systemic treatment first, then local resection” strategy.

Currently, several treatment options are available for NELMs, and surgical resection is one of them. However, the oncological characteristics and genetic backgrounds differ among these tumors, and their responses and prognoses vary. The optimal timing and strategy of resection, NELM patterns, and prognoses have been poorly investigated. Research, especially regarding long-term survival comparisons among patients with liver metastases from NETs is relatively limited. Additionally, at Hokkaido University, an observation rule has been adopted for NELMs, and the effect of this principle on patient prognosis requires corresponding clarification. Considering most NELMs originate from pancreatic NETs (P-NETs) or rectal NETs (R-NETs) [11, 12] both in China and Japan, we included this subset of patients to observe the real-world status of NET treatment for NELMs and explore the effect of the observation rule on patient prognosis.

## Methods

### Patients

Based on the current status of the databases, patient data were extracted separately from Hokkaido University Hospital (Hokkaido, Japan) between February 1, 2003, and December 31, 2022, and from Sichuan Provincial People’s Hospital Affiliated to University of Electronic Science and Technology of China (Chengdu, China) between September 1, 2018, and June 30, 2023. The inclusion criteria were as follows: (1) patients diagnosed with NEN by pathology and/or histochemical staining; (2) patients with WHO NET grades 1, 2, or 3; (3) patients with synchronous or asynchronous liver metastasis (LM); and (4) patients without perioperative death. The exclusion criteria were as follows: (1) patients with tumors that did not originate from the pancreas or rectum, (2) patients diagnosed as functional NETs, and (3) patients with other types of malignant tumors.

The clinicopathological factors of these patients were collected, including background patient data, operative details, tumor characteristics, pathological results, treatment strategies, and surgical outcomes. NELMs originating from the pancreas or rectum were included in the P-NELM and R-NELM groups.

### The definition of neuroendocrine liver metastasis (NELM) patterns and clinical characteristics

NELMs were classified into three types [13] (a) unifocal pattern/type 1, a single LM irrespective of the size and location; (b) multifocal pattern/type 2, liver metastases with one lobe primarily affected but with smaller satellite lesions in the other; and (c) diffuse pattern/type 3, multifocal liver metastases. Semiquantitative visual assessment of hepatic tumor burden (HTB) categorized as ≤ 10%, 11–25%, 26–50%, and > 50% [14] was used to evaluate the HTB according to the enhanced computed tomography and/or magnetic resonance imaging (MRI).

OS was defined as the time from pathological diagnosis to death from any cause or the last follow-up. The proportion of OS was calculated using the Kaplan–Meier method. OS after LM (OSALM) was calculated from the time of diagnosis of LM to death or the last follow-up, including patients with synchronous or asynchronous LM. The disease-free survival interval (DFS-I) was calculated from the primary tumor curative resection to the recurrence of LM (only for asynchronous patients). Disease-free survival after hepatectomy (DFS-AH) was calculated from the time of the initial curative resection of hepatic metastasis until the second recurrence of LM.

### Definition of surgical treatments

Primary tumor and LM curative-intent resection (PLCR): patients with synchronous or asynchronous liver metastases underwent simultaneous or sequential curative-intent resection of both primary and metastatic lesions.

Primary tumor radical resection (PTR): curative resection of the primary lesion followed by tumor debulking surgery for LM or not.

### Observation rules for the Japanese cohort

In the Japanese cohort, the 3-month observation rules were tried to introduced in the treatment of type 1 and 2 NELMs from 2021 [15]. Briefly, if the number of tumors remains unchanged during the 3-month observation period, regardless of introduce of chemotherapy or other system treatments, we could initially rule out the possibility that potentially implanted metastatic seed developed, radical or debulking surgery was evaluated in the multidisciplinary cancer board.

### Statistical methods

Continuous variables that conformed to normal distribution are shown as the means ± standard deviations and analyzed using the independent T-test. Otherwise, they were recorded as median (interquartile range: P25, P75) and analyzed using the Mann–Whitney *U*-test. Categorical variables were compared using Fisher’s exact test or the chi-squared test. The mean OS of different groups were estimated using the Kaplan-Meier method, and the differences were compared by the log-rank test. Variables with a p-value < 0.2 were included in multivariate analyses using a Cox proportional hazards model. All differences with a P‐value of < 0.05 were considered significant. Statistical analyses were performed using JMP 14 (SAS Institute Inc., Cary, NC, USA).

### Consent

All patients provided written informed consent prior to surgery. This study was conducted in accordance with the Declaration of Helsinki and approved by both Hokkaido University (017–0136) and Sichuan Provincial People’s Hospital (Ethics Review [Res.] No. 391, 2022).

## Results

### Patients’ characteristics

In total, 47 patients from Hokkaido University and 34 patients from Sichuan Provincial People’s Hospital were included. The final follow-up was conducted in September 2023. In total, 69 (85.2%) tumors originated from the pancreas, whereas 12 (14.78%) originated from the rectum. The enrolled patients had a median age of 56.7 (range, 33–85.2) years, and 45 of the 81 (55.6%) patients were female. Within this cohort, 11, 63, and 7 patients were classified as having WHO NET G1, G2, and G3, respectively. Synchronous NELMs were diagnosed in 53 (65.4%) patients, whereas 28 (34.6%) patients had asynchronous NELMs. The median Ki-67 level was 5.0 (range, 3.0–10.0). The median follow-up time was 76.0 months.

### Clinical features of NELMs according to the different original sites

There were no statistically significant differences in sex, Ki-67 expression, lymph node metastasis at diagnosis (LNMD), patterns of NELMs, HTB, and treatment patterns between the P- and R-NELM groups. Patients in the P-NELM group (55.41 ± 11.93) were significantly younger than those in the R-NELM group (68.03 ± 7.87) (*P* < 0.001). The distribution of primary tumor grades (WHO criteria) for P-NETs was 9:54:6, and that for R-NETs was 2:9:1 (*P* = 0.947). The proportion of synchronous NELMs was greater in the P-NELM group (69.6%, 48/69) than in the R-NELM group (41.7%, 5/12), but this difference was not statistically significant (*P* = 0.122).

In patients with P-NELMs, 43 underwent pancreatectomy, including 24 distal pancreatectomy (DP), 16 pancreaticoduodenectomy (PD), 1 DP + PD, 1 total pancreatic resection, and 1 pancreatic tumor enucleation. Among them, 19 patients underwent NELM radical resection and/or ablation. A minimum of 24 patients underwent debulking surgeries (including liver resection and/or ablation of partial metastatic lesions) and transcatheter arterial embolization (TACE)/transarterial embolization. For R-NELMs, endoscopic mucosal resection (EMR) and low anterior resection (LAR) were performed in four and five patients, respectively. For synchronous NELMs, only two patients underwent simultaneous LAR and partial hepatic resection. All patients with R-NELMs, including three with unresected primary tumors, received comprehensive therapies, such as TACE, targeted therapy, somatostatin analogs, chemotherapy, immunotherapy, and PRRT. Comprehensive therapy was also administered to 95.7% (66/69) of the patients with P-NELMs. However, three (4.3%) Chinese patients with synchronous P-NELMs refused any treatment and were consistently managed through observation throughout the entire process. The treatment patterns for P-NELMs and R-NELMs were similar (*P* = 0.164) (Table 1).

### Prognostic outcomes for NELMs

The estimated mean OS time of all patients was 165.9 months. There was no significant difference between the P-NELM and R-NELM groups in terms of OS (*P* = 0.503) and OSALM (*P* = 0.546). For further analysis of NELM prognosis, we grouped WHO G1 and G2 together to compare with G3, and compared HTB ≤ 10% with > 10%. Considering the equivalence of patient numbers and the difference in OS, we set the cutoff values for age at 60 years and the Ki-67 level at 7%.

**Table 1:** Comparison of clinical characteristics between pancreatic neuroendocrine liver metastases (NELMs) and rectum neuroendocrine NELMs

	*P*-NELMs (*n* = 69)	*R*-NELMs (*n* = 12)	χ^2^/t/Z	*P* value
Sex: female/male, n (%)	40 (58.0)/29 (42.0)	5(41.7)/7(58.3)	1.101	0.294
Age: (mean ± SD), y	55.41 ± 11.93	68.03 ± 7.87	-3.522	0.000
WHO grade: (G1/G2/G3), n (%)^*^	9 (13.0)/54 (78.3)/6 (8.7)	2(16.7)/9(75.0)/1(8.3)	0.109	0.947
Synchronous/asynchronous, n (%)	48 (69.6)/21 (36.4)	5(41.7)/7(58.3)	2.392	0.122
LNMD: Y/N, n (%)^#^	15 (21.7)/54 (78.3)	1(9.1)/10(90.9)	0.323	0.570
Ki-67 of primary tumor: ≤7/>7, n (%) ^#^	37 (54.4)/31 (45.6)	7(63.6)/4(36.4)	0.060	0.807
Pattern of NELM: type I/II/III, n (%)^#^	32 (47.1)/27 (39.7)/9 (13.2)	3(25.0)/5(41.7)/4(33.3)	3.372	0.185
HTB: ≤10%/11–25%/26–50%/>50%, n (%)^#^	35 (51.5)/18 (26.5)/10 (14.7)/5 (7.4)	5(41.7)/2(16.7)/1(8.3)/4(33.3)	5.421	0.143
Treatment patterns: n (%)			5.115	0.164
PLCR	19 (27.5)	1(8.3)		
PTR	25 (36.2)	8(66.7)		
Comprehensive therapy^&^	22 (31.9)	3(25.0)		
Observation	3 (4.3)	0(0.0)		

^*^One patient could not be staged. ^#^Only patients with data were included. ^&^TACE/TAE/PRRT/systemic chemotherapy. P-NELM, pancreatic neuroendocrine liver metastasis; R-NELM, rectal neuroendocrine liver metastases; LNMD, lymph node metastasis at diagnosis; HTB, hepatic tumor burden; WHO, World Health Organization; PLCR, primary tumor and liver metastasis curative-intent resection; PTR, primary tumor radical resection only/or with NELM debulking surgery; PRRT, peptide receptor radionuclide therapy; N, No; Y, yes

In the univariate analysis of OS for the whole cohort, there was no statistically significant difference in sex, primary tumor, age, nationality, LNMD, NELM pattern, and NELM radical resection (all *P* > 0.5). However, WHO G1 and G2, asynchronous, Ki-67 ≤ 7%, HTB ≤ 10%, and surgical treatment were associated with significantly longer OS time (all *P* < 0.05). Further comparison was conducted for OS time among the PLCR, PTR, and comprehensive therapy groups, and the results showed that only the difference between the PTR and comprehensive therapy groups was significant (estimated mean: 191.1 months vs. 83.9 months, *P* = 0.017). Although the OS time in PTR was longer than that in PLCR, the difference was not statistically significant (estimated mean: 116.4 months vs. 191.1 months, *P* = 0.051). Additionally, the comparison of OSALM between PLR and PTR results did not reveal any statistically significant difference. Considering the potential effect of lymph node metastasis on prognosis, we included this factor in the multivariate analysis. Subsequently, the results showed that synchronous NELMs, Ki-67 > 7, and HTB > 10% were independent risk factors (hazard ratio [HR] = 4.27, 95% confidence interval [CI] = 1.26–14.49, *P* = 0.020; HR = 3.31, 95% CI = 1.10–9.98, *P* = 0.034; and HR = 5.70, 95% CI = 1.28–25.34, *P* = 0.022, respectively) (Table 2).

**Table 2:** Analysis of overall survival in all patients

	Univariate analysis				Multivariate Cox regression					
		Estimated Mean ± SD	χ^2^	P value		B	P value	HR	HR 95% CI	
Sex (months)	Female	152.7 ± 16.8	0.310	0.578						
	Male	175.9 ± 19.0								
Primary tumor	Pancreas	155.0 ± 14.7	0.449	0.503						
	Rectum	179.4 ± 24.6								
Age (y)	≤ 60	162.8 ± 16.9	0.113	0.737						
	>60	161.6 ± 19.2								
Nationality	Japanese	170.9 ± 14.0	0.385	0.535						
	Chinese	106.6 ± 14.1								
WHO grade^*^	G1 + G2	169.6 ± 13.2	5.116	0.024	G1 + G2					
	G3	51.1 ± 13.1			G3	1.44	0.135	4.20	0.64	27.53
Metastatic pattern	Synchronous	111.9 ± 14.3	6.076	0.014	Synchronous	1.45	0.020	4.27	1.26	14.49
	Asynchronous	193.6 ± 14.3								
LNMD	N	169.1 ± 14.3	0.777	0.378	N					
	Y	100.3 ± 15.8			Y	0.16	0.814	1.18	0.30	4.60
Ki-67 of original tumor	≤ 7	196.7 ± 13.6	6.601	0.010	≤ 7					
	> 7	95.6 ± 11.7			> 7	1.20	0.034	3.31	1.10	9.98
Pattern of NELM^#^	Type I	175.2 ± 18.1	1.883	0.390						
	Type II	148.3 ± 18.9								
	Type III	151.2 ± 30.5								
HTB^#^	≤ 10%	192.3 ± 14.3	7.283	0.007	≤ 10%					
	> 10%	137.9 ± 18.5			> 10%	1.74	0.022	5.70	1.28	25.34
Surgical treatments^^^	Y	174.6 ± 13.7	4.036	0.045	N	0.65	0.401	1.91	0.42	8.66
	N	86.3 ± 11.2								
Treatment Methods	PLCR	116.4 ± 18.0	3.795	0.051^1^						
	PTR	191.1 ± 15.0	0.202	0.653^2^						
	Comprehensive Therapy	83.9 ± 12.0	5.730	0.017^3^						

^*^One patient could not be staged. ^#^Only patients with data were included. ^&^Including two cases of TACE/TAE and partial liver resection. ^^^Includes PLCR and PTR (PLCR, primary tumor and liver metastasis curative-intent resection; PTR, primary tumor radical resection and LM debulking surgery)

^1^P-value of OS between PLCR and PTR;^2^P-value of OS between PLCR and comprehensive therapy;^3^P-value of OS between PTR and comprehensive therapy

NELM, neuroendocrine liver metastasis; HR, hazard ratio; HTB, hepatic tumor burden; LNMD, Lymph node metastasis at diagnosis; N, No; Y, yes

Univariate analysis identified WHO G3, Ki-67 > 7%, and HTB > 10% as positive factors for P-NELMs (Table 3). In the multifactorial analysis, considering the correlation between the NELM pattern and HTB and the effect of metastasis pattern and surgical treatments on prognosis in all patients, we opted to exclude the NELM pattern and included the WHO G stage, metastasis pattern, Ki-67 level, HTB, and surgical treatments in the multivariate analysis for a more comprehensive examination. The results revealed that only WHO G3 and HTB > 10% were independent risk factors (HR = 6.64, 95% CI = 1.05–42.24, *P* = 0.043 and HR = 12.45, 95% CI = 1.58–98.44, *P* = 0.017, respectively) (Table 3). However, based on different sources, univariate analysis for R-NELMs suggested that synchronous NELMs and without surgical treatments could be risk factors, but no independent risk factors were found during the multivariate analysis.

**Table 3:** Analysis of overall survival for pancreatic neuroendocrine liver metastases

	Univariate analysis				Multivariate Cox regression					
		Estimated mean ± SD	χ^2^	P value		B	P value	HR	95% CI	
Sex	Female	154.5 ± 18.7	0.002	0.961						
	Male	135.4 ± 16.4								
Age	≤ 60	162.8 ± 18.2	0.496	0481						
	> 60	104.9 ± 12.6								
Nationality	Japanese	158.5 ± 15.7	0.078	0.780						
	Chinese	120.1 ± 8.6								
WHO G grade^*^	G1 + G2	159.3 ± 14.9	6.013	0.014	G3	1.89	0.045	6.64	1.05	42.24
	G3	45.2 ± 14.9								
Metastatic pattern	Synchronous	115.3 ± 16.4	2.656	0.103	Synchronous	0.73	0.303	2.07	0.52	8.22
	Asynchronous	178.0 ± 18.0								
LNMD	N	160.1 ± 16.4	0.613	0.434						
	Y	99.3 ± 16.0								
Ki-67 of primary tumor^#^	≤ 7	185.8 ± 15.6	4.541	0.033						
	> 7	93.0 ± 13.6			> 7	0.57	0. 387	1.77	0.49	6.42
Pattern of NELM^#^	Type I	174.7 ± 21.4	5.384	0.068						
	Type II	126.8 ± 15.8								
	Type III	72.4 ± 21.1								
HTB^#^	≤ 10%	193.2 ± 16.6	8.909	0.003	> 10%	2.52	0.017	12.45	1.58	98.44
	> 10%	103.6 ± 15.7								
Surgical treatment^^^	N	135.9 ± 18.6	2.063	0.151						
	Y	144.2 ± 7.4			Y	-1.264	0.235	0.28	0.04	2.28

*One patient could not be staged. ^#^Only patients with data were included. ^^^Includes PLCR and PTR (PLCR, primary tumor and liver metastasis curative-intent resection; PTR, primary tumor radical resection and LM debulking surgery). HTB, hepatic tumor burden; B, regression coefficients; HR, hazard ratio; N, No; Y, yes

In the context of various metastatic patterns, both univariate and multivariate analyses revealed that WHO G3 was an independent risk factor for patients with synchronous NELMs (HR = 11.56, 95% CI = 1.61–83.19, *P* = 0.015). Conversely, patients with asynchronous NELMs did not exhibit any significant risk factors in either univariate or multivariate analysis. Although the prognosis for patients with asynchronous NELMs did not show notable differences, a comparison of characteristics among patients from different sources indicated a significantly smaller proportion of cases with type III NELMs in P-NELMs compared with R-NELMs (*P* = 0.042). There were no statistically significant differences in the OS and OSALM between the two groups. However, the median DFS-I for P-NELMs (38.6 months) was significantly shorter than that for R-NELMs (121.0 months), and 71.4% of the R-NELMs were classified as G2 stage (*P* < 0.001) (Table 4).

**Table 4:** Comparison of clinical characteristics of asynchronous neuroendocrine liver metastases

	*P*-NELMs (*n* = 21)	*R*-NELMs (*n* = 7)	χ^2^/ Z	*P* value
Sex: female/male, n (%)	14 (66.7)/7 (33.3)	3 (42.9)/4 (57.1)	0.381	0.249
Age: ≤60/>60, n (%)	12 (57.1)/9 (42.9)	2 (28.6)/5 (71.4)	0.385	0.192
Nationality: Japanese/Chinese, n (%)	15 (71.4)/6 (28.6)	6 (85.7)/1 (14.3)	0.063	0.801
WHO grade: (G1/G2/G3), n (%)^*^	5 (23.5/15 (71.4)/1 (4.8)	2 (28.6)/5 (71.4)/0 (0.0)	0.622	0.733
LNMD: Y/N, n (%)	2 (9.5)/19 (90.5)	0 (0.0)/7 (100.0)	1	0.556
Ki-67 of the primary tumor: ≤7/>7, n (%)^#^	13 (65.0)/7 (35.0)	5 (71.4)/2 (28.6)	1	0.571
Pattern of NELM: Type I/II/III, n (%)^#^	12 (57.1)/7 (33.3)/2 (9.5)	2 (28.6)/1 (14.3)/4 (57.1)	6.341	0.042
HTB: ≤10%/>10%, n (%)^#^	14 (66.7)/7 (33.3)	3 (42.9)/4 (57.1)	0.381	0.249
Treatment patterns: n (%)			4.757	0.093
PLCR	6 (28.6)	0 (0.0)		
PTR	14 (66.7)	7 (100.0)		
Comprehensive treatment^&^	1 (4.8)	0 (0.0)		
Observation	0 (0.0)	0 (0.0)		
OS, estimated mean ± SD, months	177.9 ± 18.0	212.8 ± 15.8	0.605	0.437
OSALM, estimated mean ± SD, months	128.2 ± 18.1	51.9 ± 1.5	0.020	0.886
DFS-I, estimated mean ± SD, months	38.7 ± 28.7	135.3 ± 55.4	-4.121	0.007
DFS-I, median (P25, P75), months	38.6 (15.5–53.8)	121.0 (97.3–193.5)	–3.383	< 0.001

^*^One patient could not be staged. ^#^Only patients with data were included. ^&^Including two cases of TACE/TAE and partial liver resection. NELMs, neuroendocrine liver metastases; P-NELMs, pancreatic neuroendocrine liver metastases; R-NELMs, rectal neuroendocrine liver metastases; WHO, World Health Organization; LNMD, lymph node metastasis at diagnosis; HTB, hepatic tumor burden; PLCR, primary tumor and liver metastasis curative-intent resection; PTR, primary tumor radical resection and LM debulking surgery; OS, overall survival; OSALM, overall survival after liver metastasis; DFS-I, disease-free survival interval between the initial curative resection and recurrence; N, No; Y, yes

### Clinical features of NELMs according to different countries

Between Japanese and Chinese nationalities, the Japanese cohort exhibited a significantly higher proportion of female patients than the Chinese cohort (66.0% vs. 41.2%, *P* = 0.027). Furthermore, there was a greater likelihood of asynchronous NELMs (44.7% vs. 20.6%, *P* = 0.024) and a higher prevalence of type III LM pattern (23.4% vs. 6.1%, *P* = 0.020) in the Japanese cohort than in the Chinese cohort. Among the Japanese cohort, 36 patients were included in the observation rules; only one was ruled out after 3 months, and the remaining underwent corresponding surgical treatment. Additionally, the treatment patterns in the two countries significantly differed (*P* = 0.043). However, in terms of prognosis, both OS and OSALM showed no statistically significant differences between the two cohorts, whether considering the entire population or specifically in the subgroup of patients who underwent surgical treatment (all *P* > 0.05) (Table 5).

To further validate the utility of the observation rules, we performed a comparative analysis of the relevant data for patients undergoing PLCR in the two countries. After excluding one perioperative death and one patient with R-NELMs, we compared the remaining 18 patients with P-NELMs. There was no difference in recurrence rates between the two countries during the follow-up period. However, considering the difference in follow-up duration between the two groups, we further compared the recurrence times in patients who had already experienced recurrence. Throughout the follow-up period, seven cases of recurrence were observed in the Japanese group compared with four cases in the Chinese group. The synchronous NELM rates were 71.4% (5:2) and 75% (3:1), respectively. Comparing the median time of DFS-AH among the 11 patients, the Japanese cohort experienced a longer median recurrence time than the Chinese cohort (15.8 months vs. 6.8 months, *P* = 0.047) (Table 5).

**Table 5:** Comparison of clinical characteristics of different hospitals

	J-NELM (*n* = 47)	C-NELMs (*n* = 34)	χ2/Z	*P* value
Sex, male/female, n (%)	16(34.0)/31(66.0)	20 (58.8)/14 (41.2)	4.907	0.027
Age, ≤ 60/>60, n (%)	25(53.2)/22(46.8)	24 (70.6)/10 (29.4)	2.498	0.114
Primary tumor, pancreas/rectum, n (%)	37(78.7)/10(21.3)	32 (94.1)/2 (5.9)	3.705	0.054
WHO grade (G1/G2/G3), n (%)*	6(12.8)/38(80.9)/3(6.4)	5 (14.7)/25 (73.5)/4 (11.8)	0.841	0.657
Metastatic pattern, synchronous/asynchronous, n (%)	26(55.3)/21(44.7)	27 (79.4)/7 (20.6)	5.063	0.024
LNMD, Y/N, n (%)^#^	35(76.1)/11(23.9)	28 (82.4)/6 (17.6)	0.459	0.498
Ki 67, ≤ 7/>7, n (%) ^#^	28(60.9)/18(39.1)	16 (48.5)/17 (51.5)	1.194	0.274
Pattern of NELM, Type I/II/III, n (%) ^#^	15(31.9)/21(44.7)/11(23.4)	20 (60.6)/11 (33.3)/2 (6.1)	7.861	0.020
HTB, 1/2/3/4, n (%) ^#^	21(44.7)/11(23.4)/7(14.9)/8(17.0)	19 (57.6)/9 (27.3)/4 (12.1)/1 (3.0)	4.864	0.182
Treatment patterns, n (%)			8.171	0.043
PLCR	12(25.5)	8 (23.5)		
PTR	23(48.9)	10 (29.4)		
Comprehensive treatment	12(25.5)	13 (38.2)		
Observation	0(0.0)	3 (8.8)		
OS of the total patients, estimated mean ± SD, months	170.9 ± 14.0	106.6 ± 14.1	0.385	0.535
OSALM of the total patients, estimated mean ± SD, months	124.6 ± 12.5	52.1 ± 3.2	0.633	0.426
OS of surgical treatments^, estimated mean ± SD, months	172.5 ± 14.0	97.2 ± 13.0	2.977	0.084
OSALM of surgical treatments^, estimated mean ± SD, months	177.3 ± 14.6	114.8 ± 14.0	0.150	0.699
Recurrence of PLCR^#^, Y/N	7(70.0)/3(30.0)	4 (50.0)/4 (50.0)	0.630	0.352
DFS-AH#, median (P25, P75), months	15.8(15.4, 34.5)	6.8 (5.6–9.7)	-1.998	0.047

^*^One patient could not be staged. ^#^ Only patients with data were included. ^^^Includes PLCR and PTR (PLCR, primary tumor and liver metastasis curative-intent resection; PTR, primary tumor radical resection and LM debulking surgery). OS, overall survival; OSALM, overall survival after liver metastasis.

Disease-free survival after hepatectomy (DFS-AH): the duration between the time of initial curative resection of hepatic metastasis and recurrence of liver metastasis for the second time. LNMD, lymph node metastasis at diagnosis; NELM, neuroendocrine liver metastasis; HTB, hepatic tumor burden; N, No; Y, yes

## Discussion

NENs originate from various endocrine organs throughout the body, and different grades and primary sites have different molecular characteristics and clinical heterogeneities [16]. NENs originating from the pancreas and rectum are widely recognized as major contributors, particularly in Asian countries [17, 18]. The 5-year OS rates are 70–80% [19] for NF-P-NETs and 85.4% for colorectal-NETs [20], which are in accordance with our data of 85.1% and 80.0%, respectively. In contrast to the United States [1], P-NENs have been categorized into a better prognosis group and R-NENs into a worse prognosis group in Spanish registry data [21], detailed distinctions in clinical features, especially the characteristics of NELM between these two types, remain unclear.

Surgery is usually indicated for ENETS type I and II NELMs by clinicians, either in synchronous or asynchronous cases. However, whether resection of liver metastases improves prognosis remains controversial. Some studies suggest that primary tumor resection in patients with synchronous NELMs can result in a longer survival time, but radical surgery for both primary tumors and NELMs is not related to a better survival benefit [22, 23]. Some others: although recurrences were frequent, when combined with a multidisciplinary treatment, surgical resection should be the first option for patients with resectable NELMs [11, 24]. There are also studies reporting: good candidates (Ki-67 < 5.0% and tumor number < 7) for resection of NELMs originating from P-NETs could have longer relapse-free survival [25]. The present study delivered data supporting the first hypothesis. Although surgical treatments were not identified as independent risk factors in all patients, those who underwent surgery (including PLCR and PTR) showed an association with improved OS compared with those who did not. Additionally, there was no significant difference in the prognosis between patients undergoing PLCR and PTR. This suggests that radical surgery for NELMs may not be necessary. Especially for patients with unresectable NELM. PTR should be enough.

In patients with asynchronous NELMs, although there were no statistically significant differences in OS and OSALM, DFS-I was notably longer in patients with R-NELMs than in those with P-NELMs. Additionally, the HTB was higher in the R-NELM group than in the P-NELM group. We hypothesize that the delayed diagnosis of R-NEN recurrence may be due to infrequent post-treatment follow-ups or checks. The 2023 ENETS guidelines recommend follow-up intervals for radical treatment in differentiated G1/G2 NETs, with a suggested frequency of 6 months of MRI and annual sigmoidoscopy for at least 5 years for R-NEN [20] and every 6–12 months of MRI for 5 years, then every 12–24 months for 10 years, and then every 5 years for P-NEN [19]. However, in our study, the estimated mean OS time of asynchronous R-NELMs was 212.8 months, and the estimated mean DFS-I was nearly 135 months. For asynchronous R-NELMs, irregular follow-up beyond 5 years may lead to delayed NELM diagnosis and increased tumor burden. Then, extended and more frequent regular follow-ups are necessary, surpassing the 5-year timeframe recommended by guidelines. According to our data, 71.4% of the R-NELM cases were G2. We suggest distinguishing G2 from G1 during clinical follow-up, and that G2 should be followed up with longer follow-up periods and shorter intervals than G1.

Our data revealed comparable OS between P-NELMs and R-NELMs, but the OSALM for R-NELMs was shorter than for P-NELMs (123.25 ± 12.73 months vs. 49.50 ± 2.38 months), although without statistical significance. Possible reasons include the shorter DFS-I duration for P-NELMs (mean: 38.7 months), aligning with the recommended follow-up intervals [19]. Therefore, regular follow-up is beneficial for the timely detection and reduced incidence of type III NELMs at the time of diagnosis. Moreover, combining TACE with other treatments demonstrates superiority in P-NELMs compared with R-NELMs [26]. Conversely, R-NELMs, often treated with early-stage EMR (44.4% in our dataset), showed the potential for long-term recurrence, possibly associated with factors, such as intraoperative residue. The estimated mean DFS-I duration for R-NELMs exceeded the guidelines [19], suggesting the likelihood of more cases with type III pattern NELMs in follow-up. Hence, these factors may also play a role in categorizing P-NELMs into a better prognosis group and R-NELMs into a worse prognosis group.

Further analysis was performed to compare the prognoses of surgical treatments between the two countries, considering that observation rules were adopted in the Japanese cohort. The results indicated that the estimated mean of the Japanese cohort seemed longer than that of the Chinese cohort both in OS and OSALM of surgically treated patients, but no statistical difference was found. One significant factor that cannot be overlooked is that NETs, which are indolent tumors, have a low likelihood of developing new metastatic lesions within a 3-month period, and the probability of accurately diagnosing such lesions is minimal. Simultaneously, the inclusion period for the two countries differed, as did the corresponding follow-up durations, which has implications for survival comparisons. Therefore, we focused only on patients undergoing PLCR. The recurrence rate of the patients undergoing PLCR was the same; however, the duration of DFS-AH was longer in the Japanese cohort than in the Chinese cohort. It appears that the DFS-AH was extended to patients following observation rules, which was based on previous experience in the treatment of metastatic malignant tumors [15, 27]. We hypothesized that this principle would allow for timely resection of more potential lesions. However, given the rarity of the relevant cases, this conclusion requires further support from additional data.

The limitations of this study include its retrospective nature, temporal differences in case inclusion, and small cohort size, especially that of the R-NELM cohort. Further investigation is required to address these limitations.

In conclusion, patients with P-NETs are more likely to exhibit synchronous NELMs, whereas those with R-NETs have a higher tendency for asynchronous NELMs and a greater likelihood of type III NELMs. Therefore, in R-NELMs, extended follow-up times and shorter follow-up intervals are recommended beyond existing guidelines for patients with G2 NETs. Compared to comprehensive treatment, surgical treatment (including PLCR and PTR) is associated with improved OS. But for those combined with NELMs, PTR may be enough. The observation rules for NELMs appear to be followed by a longer interval between the first time of curative hepatectomy and the recurrence of LM. Additional cases with extended follow-up periods are required for further validation. On the basis of these characteristics, a multimodal treatment strategy should be developed, which includes local treatments such as PLCR, PTR or TACE, systemic treatments such as targeted therapy, immunotherapy or chemotherapy, and even adopting observation rules for some special patients.

## Data Availability

No datasets were generated or analysed during the current study.
